# Including ratio traits in multiple trait selection

**DOI:** 10.1186/s12711-026-01111-3

**Published:** 2026-11-11

**Authors:** Julius H. J. van der Werf

**Affiliations:** School of Environmental and Rural Science, University of New England, Armidale, NSW 2351 Australia

## Abstract

**Background:**

Animal breeding programs aim to improve productivity while reducing input costs and resource use. As sustainability has become a central concern, feed intake and enteric methane emissions now feature more prominently in breeding objectives, supported by increasing availability of individual-animal measurements for those traits. An important question is how to incorporate these traits most effectively in selection indices that aim at improving both resource efficiency and environmental outcomes. A seemingly appealing strategy is to augment existing selection indices with a ratio trait such as production per unit of feed intake or methane emitted per unit of product, as direct indicators of efficiency. This paper will investigate selection indices that include a ratio trait. Selection index theory was used to compare single trait selection for a ratio trait with selection based on a linear index comprising the component traits, as well as with selection including both the ratio trait and one of its components. Genetic parameters for ratio traits were approximated from those of the component traits. When incorporating a ratio trait into the breeding objective, the economic weights of the component traits were correspondingly adjusted.

**Results:**

When ratio traits are used as a single trait selection criterion, the response to selection is driven largely by the coefficient of genetic variation of each component trait. However, an index including a ratio trait as well as one of the component traits is equivalent to a linear index with the two component traits. A breeding objective including both a ratio trait and a component trait requires an adjustment of economic values of the component traits.

**Conclusion:**

Ratio traits such as feed efficiency and methane intensity can be used in selection indices as they give equivalent selection responses as indices that include the component traits (e.g. feed intake and methane production) alongside production traits, provided that at least one of the component traits—either the numerator or denominator—is also included in the index and economic weights are adjusted accordingly.

## Background

Animal breeding programs have traditionally aimed to improve productivity by maximizing product output relative to input costs and resource use. Improving feed efficiency—minimizing feed use relative to product output—has long been a central breeding objective, particularly in intensive livestock industries where individual feed intake recording has been established for several decades [1]. In ruminant livestock, feed intake measurement has gained renewed importance beyond production efficiency, driven by growing emphasis on environmental sustainability and the need to reduce the carbon footprint of animal agriculture. Reducing enteric methane emissions has emerged as an explicit breeding objective, and methane production is strongly associated with feed intake. With the growing availability of individual phenotypic data for feed intake and methane emissions in sheep and cattle, the question of how to most effectively incorporate these traits into selection indices has become increasingly important.

In an economic sense, methane is like feed intake in that it is a cost to the system, likely phenotypically and genetically positively correlated to animal productivity. There are several variables that can define resource use efficiency. Assume a breeding objective that values increase in production and a decrease in resource use. With regard to feed intake, the options for selection for increased resource efficiency include (1) using a linear selection index with a positive economic value for production and a negative value for feed intake (2) selecting directly for a simple measure of feed efficiency, i.e. selecting to increase a ratio of production divided by feed intake (3) selecting directly for feed conversion, i.e. selecting to lower the ratio of feed intake divided by production, which is simply the reverse of (2), and (4) selecting for production and residual feed intake, i.e. feed intake adjusted for the feed required for production. With selection for methane, similar options for selection have been suggested, e.g. selecting for lower methane production per head, selection for lower methane intensity (methane production per unit of production output), methane yield (methane production per unit of feed intake) and residual methane (methane output adjusted for the methane needed for production). It has been documented before that an index with residual traits can be equivalent to an index with the unadjusted component traits that includes the correlation between these traits, provided that the economic values are appropriately adjusted [2, 3]. This paper will mainly focus on theoretical aspects of including ratio traits in selection indices.

Several papers have considered selection on a ratio trait and considered several statistical problems [4–6]. Lin et al. [4], showed that when the ratio of component traits is a breeding objective, it implies a weighting on the component traits proportional to the means of the traits, rather than to their economic values. In a simulation study, Gunsett [5] found that selection on a ratio of traits is less efficient than including the component traits directly in a linear index. Based on a result by Sutherland [7], Veerkamp and Emmans [6] concluded that with single trait selection on a ratio, the response depends on the ratios of coefficients of variation and heritabilities of the component traits. None of these studies explored the properties of a ratio trait when included in a multiple trait selection index, which may also include at least one of the component traits. This is relevant as most breeding programs have included ratio traits such as feed conversion or feed efficiency and selected for those alongside one of the component traits, usually growth.

The aim of this paper is to explore the properties of ratio traits and how ratio trait parameters depend on the parameters of the component traits. Selection index theory will be used to predict selection responses for both single trait and multiple trait selection strategies. An example of feed intake and growth in beef cattle, and feed conversion ratio as the ratio trait will be used as a case study.

## Materials and methods

### Deriving the phenotypic and genetic variance for a ratio trait

We will derive an analytical procedure to predict response to selection when using ratios and explore their dependency on key parameters. Some of this was also presented by Sutherland [7], but this paper will aim for a more general and concise approach.

Assume a ratio of two phenotypes of component traits, x and y, with means μ_x_ and μ_y_, and phenotypic variances $\sigma _x^2$ and $\sigma _y^2$. Using a Taylor series approximation, Lynch and Walsh [8] showed that the variance of the ratio is

$${\sigma ^2}\left({{x \over y}} \right) = \sigma _r^2 = {\left({{{{\mu _x}} \over {{\mu _y}}}} \right)^2}\left({{{\sigma _x^2} \over {\mu _x^2}} - {{2{\sigma _{xy}}} \over {{\mu _x}{\mu _{xy}}}} + {{\sigma _y^2} \over {\mu _y^2}}} \right),$$

where ${\sigma _{xy}}$ is the covariance between x and y.

The phenotypic variance of a ratio of two component traits can thus be written as a function of the coefficients of variation $v{p_i} = {{\sigma {p_i}} \over {{\mu _i}}}$ and the phenotypic correlation (r_p_) between the component traits.

1 $$\sigma _p^2\left( {\frac{x}{y}} \right) = \sigma _{p.r}^2 = {\left( {\frac{{{\mu _x}}}{{{\mu _y}}}} \right)^2}(vp_x^2 - 2{r_p}v{p_x}v{p_y} + vp_y^2)$$

The phenotypic variance of the ratio trait ($\sigma _{p.r}^2$) depends therefore on the squared value of the ratio of trait means ${\left({{{{\mu _x}} \over {{\mu _y}}}} \right)^2}$, the coefficient of phenotypic variation of each component trait and the phenotypic correlation between the two component traits. The variance is higher with a lower phenotypic correlation between the component traits.

Similarly, we can derive the genetic variance of the ratio by using coefficients of genetic variation ($$v{a_i} = {{\sigma {a_i}} \over {{\mu _i}}} = {{{h_i}\sigma {p_i}} \over {{\mu _i}}} $$=$$ {h_i}v{p_i}$$), and the genetic correlation between the component traits ($${r_g}$$), where $$h_i^2$$ is the heritability of trait i.

2 $$\sigma _a^2\left({{x \over y}} \right) = \sigma _{a.r}^2 = {\left({{{{\mu _x}} \over {{\mu _y}}}} \right)^2}(va_x^2 - 2{r_g}v{a_x}v{a_y} + va_y^2)$$

Therefore, the additive genetic variance of the ratio trait ($\sigma _{a.r}^2$) depends linearly on the squared value of the ratio of trait means ${\left({{{{\mu _x}} \over {{\mu _y}}}} \right)^2}$ and the coefficient of genetic variation of each trait as well as the genetic correlation. The genetic variance is higher with a lower genetic correlation between the component traits.

The genetic correlation does not affect the phenotypic variance of the ratio trait, and the phenotypic correlation does not affect the genetic variance.

### Heritability of a ratio trait

The heritability of the ratio trait is $h_r^2 = \sigma _a^2\left({{x \over y}} \right)/\sigma _p^2\left({{x \over y}} \right)$ is therefore the ratio of the terms in Eqs. (1) and (2):

3 $$ h_r^2 = {{va_x^2 - 2{r_g}v{a_x}v{a_y} + va_y^2} \over {vp_x^2 - 2{r_p}v{p_x}v{p_y} + vp_y^2}} = {\rm{ }}{{\left({\lambda _a^2 - 2{r_g}{\lambda _a} + 1} \right)} \over {\left({\lambda _p^2 - 2{r_p}{\lambda _p} + 1} \right)}}h_y^2$$

where ${\lambda _p}$ is the ratio of coefficients of phenotypic variation of traits x and y (${\lambda _p} = v{p_x}/v{p_y}$) and λ_a_ = $v{a_x}/v{a_y}$ is the ratio of coefficients of genetic variation. This result is similar to the result by Sutherland [7].

The heritability therefore depends on the coefficients of variation ($v{p_i}$) of the component traits, the heritability of the component traits, and the genetic and phenotypic correlation between traits x and y. The heritability is not affected by the trait means and is therefore independent of the units of measure of traits x and y.

**Fig. 1:**
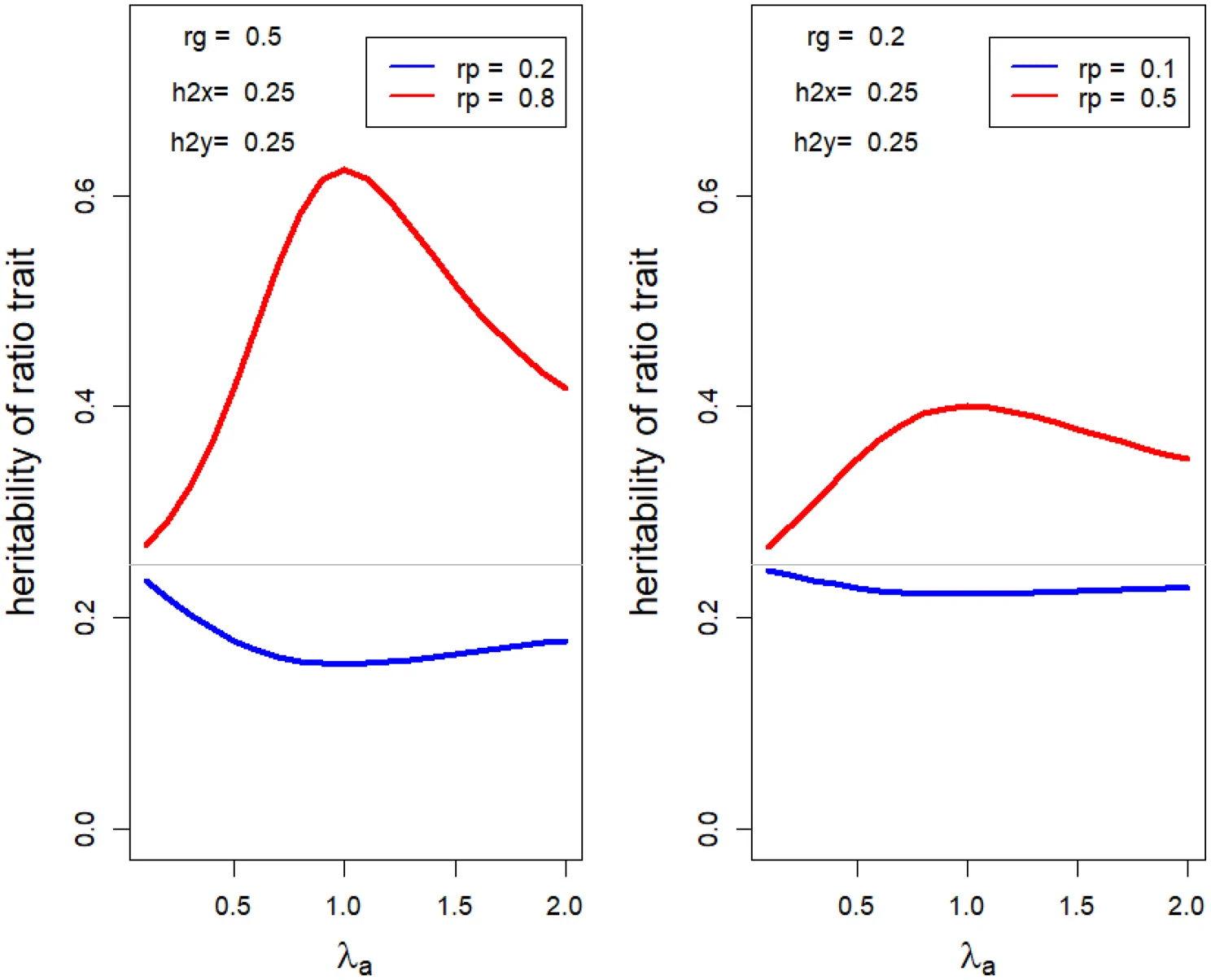
Heritability of a ratio trait as a function of the ratio of the coefficient of genetic variation λ_a_ with equal heritability of the component traits

The heritability of the ratio is equal to the heritability of the component traits if these are equal and if the phenotypic and genetic correlations are equal. Values of heritability of the ratio trait as a function of λ_a_ are shown in Fig. 1. The phenotypic correlation has a large effect on $h_r^2$, which is higher if r_p_ > r_g_ and it is lower if r_p_ < r_g_. For example, with component trait heritabilities of 0.25, the ratio trait could have a heritability of 0.6, when r_g_ = 0.5 and r_p_ = 0.8. Eqs. (1) and (2) show that the variances will be low with high correlations. When r_p_ is larger than r_g_, the phenotypic variance is relatively more reduced than the genetic variance, leading to a higher heritability.

Since r_p_ is bounded by the value of r_g_ and the heritability values, it is insightful to look at the values of r_g_ and r_e_ instead, where r_e_ is the correlation between the environmental effects of the component traits. When r_g_ – r_e_ is large and positive, the heritability of the ratio traits will be very low, whereas with large negative values for r_g_ – r_e_, the heritability will be very high

**Fig. 2:**
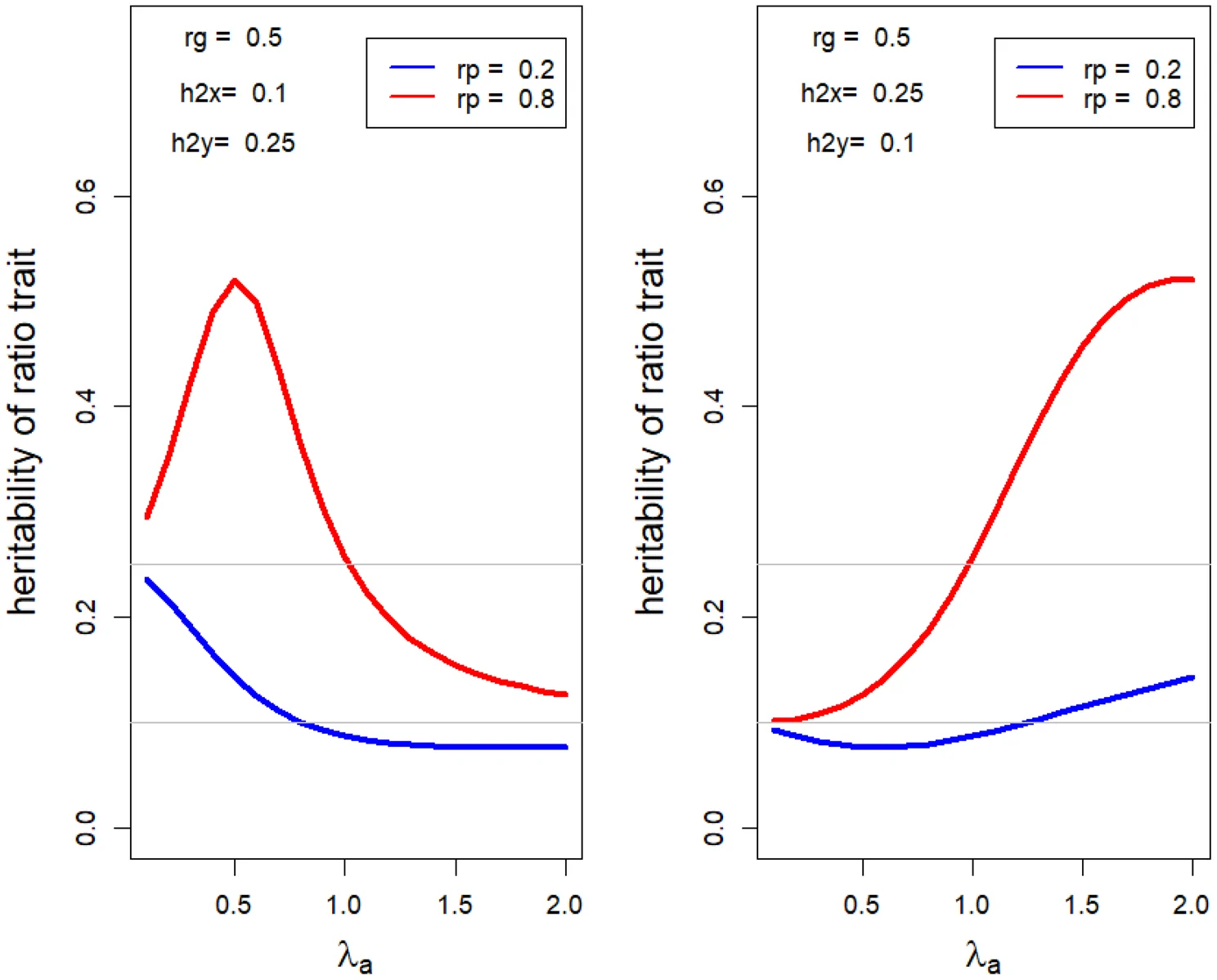
Heritability of a ratio trait as a function of the ratio of the coefficient of genetic variation λ with unequal heritability of the component traits

When the heritabilities of the component traits differ, then the ratio trait heritability will be higher for lower lambda values (λ_a_ < 1) if the denominator trait (y) has higher heritability, and $h_r^2$ will be higher for higher value of lambda (λ_a_ > 1) if the numerator trait (x) has a higher heritability (Fig. 2).

### Phenotypic and genetic correlation of a ratio trait with its component traits

The correlation between the ratio trait and the component traits can be derived using the approximation by Pearson [10] as cited by Sutherland [7]. The correlation between two ratios is approximately

4 $$\begin{aligned} & {r_{\frac{{{x_1}}}{{{x_3}}},\frac{{{x_2}}}{{{x_4}}}}} \cong \\ & \frac{{{r_{{x_1}{x_2}}}{v_{{x_1}}}{v_{{x_2}}} - {r_{{x_1}{x_4}}}{v_{{x_1}}}{v_{{x_4}}} - {r_{{x_2}{x_3}}}{v_{{x_2}}}{v_{{x_3}}} + {r_{{x_3}{x_4}}}{v_{{x_3}}}{v_{{x_4}}}}}{{{{\left( {v_{{x_1}}^2 + v_{{x_3}}^2 - 2{r_{{x_1}{x_3}}}{v_{{x_1}}}{v_{{x_3}}}} \right)}^{^1{/_2}}}{{\left( {v_{{x_2}}^2 + v_{{x_4}}^2 - 2{r_{{x_2}{x_4}}}{v_{{x_2}}}{v_{{x_4}}}} \right)}^{^1{/_2}}}}} \\ \end{aligned} $$

where r_ij_ is the correlation between variables i and j and $$ {v_i}$$ is the coefficient of variation for variable i (= σ_i_/μ_i_). For example, for the phenotypic correlation between ratio trait x/y and component trait x, we replace the variables for *x*_*1*_, *x*_*2*_, *x*_*3*_ and *x*_*4*_ by x, x, y and 1, respectively such that

5 $$\begin{aligned} {r_{p\frac{x}{y},x}} & \cong \frac{{vp_x^2 - {r_p}v{p_x}v{p_y}}}{{{{\left( {vp_x^2 + vp_y^2 - 2{r_p}v{p_x}v{p_y}} \right)}^{^1{/_2}}}{{\left( {v_x^2} \right)}^{^1{/_2}}}}} \\ & = \frac{{v{p_x} - {r_p}v{p_y}}}{{{{\left( {vp_x^2 + vp_y^2 - 2{r_p}v{p_x}v{p_y}} \right)}^{^1{/_2}}}}} \\ & = \frac{{\left( {{\lambda _p} - {r_p}} \right)}}{{{{\left( {\lambda _p^2 + 1 - 2{r_p}{\lambda _p}} \right)}^{^1{/_2}}}}} \\ \end{aligned} $$

where r_p_ is the phenotypic correlation between the component traits. To find the phenotypic correlation between ratio trait x/y and trait y, we replace the variables for *x*_*1*_, *x*_*2*_, *x*_*3*_ and *x*_*x*_ by x, y, y and 1, respectively such that

6 $$\begin{aligned} {r_{p\frac{x}{y},y}} & \cong \frac{{{r_p}v{p_x} - v{p_y}}}{{{{\left( {vp_x^2 + vp_y^2 - 2{r_p}v{p_x}v{p_y}} \right)}^{^1{/_2}}}}} \\ & = \frac{{ - \left( {v{p_y} - {r_p}v{p_x}} \right)}}{{{{\left( {vp_x^2 + vp_y^2 - 2{r_{xy}}v{p_x}v{p_y}} \right)}^{^1{/_2}}}}} \\ & = \frac{{\left( {{\lambda _p}{r_p} - 1} \right)}}{{{{\left( {\lambda _p^2 + 1 - 2{r_p}{\lambda _p}} \right)}^{^1{/_2}}}}} \\ \end{aligned}$$

These results are equal to those of Sutherland [7], with a slightly different notation. The phenotypic correlation between the ratio trait and component trait x is generally positive while the correlation with component trait y is mostly negative. With equal $$v{p_x}$$ and $$v{p_y}$$, $${\lambda _p}$$ = 1 and the value of $${r_{p{x \over y},x}}$$ is always equal, but of opposite sign, to $${r_{p{x \over y},y}}$$, and the absolute value of these correlations is larger as $${r_{xy}} $$ is closer to zero.

The phenotypic correlation between the ratio trait and trait x is lower when $$v{p_x} $$ < $$ v{p_y}$$, i.e. λ_p_ < 1, and higher with $$v{p_x} $$>$$ v{p_y}$$ (λ_p_ > 1) and the correlation with trait y is more negative when λ_p_ < 1 and less negative or possibly positive with λ_p_ > 1. When $$ v{p_x}$$ >> $$v{p_y}$$, then $${r_{p{x \over y},x}}$$ approaches 1, and when $$ v{p_x}$$ << $$ v{p_y}$$ the correlation $${r_{p{x \over y},y}}$$ approaches * - 1.* The ratio of $$ {\mu _x}$$/$$ {\mu _y}$$ has no effect on these phenotypic correlations.

For deriving the genetic correlation between the ratio trait and each of the component traits, we use Eq. (5) and Eq. (6) but replace the phenotypic correlation r_p_ by the genetic correlation ${r_g}$ and the ratio of coefficients of variation ${\lambda _p}$ with the ratio of coefficients of genetic variation ${\lambda _a}$:

7 $$\begin{aligned}r_{g_{\frac{x}{y},x}}&\simeq\frac{v a_x^2-r_g v a_x v a_y}{(v a_x^2+v a_y^2-2r_g v a_x v a_y)^{^1{/_2}}(v a_x^2)^{^1{/_2}}}\\[6pt]&=\frac{v a_x-r_g v a_y}{(v a_x^2+v a_y^2-2r_g v a_x v a_y)^{^1{/_2}}}\\[6pt]&=\frac{(\lambda_a-r_g)}{(\lambda_a^2+1-2r_g\lambda_a)^{^1{/_2}}}\end{aligned}$$

8 $$\begin{aligned}r_{g_{\frac{x}{y},y}}&\simeq\frac{r_g v a_x-v a_y}{\left(v a_x^2+v a_y^2-2r_g v a_x v a_y\right)^{^{1/_{2}}}}\\[6pt]&=\frac{-(v a_y-r_g v a_x)}{\left(v a_x^2+v a_y^2-2r_g v a_x v a_y\right)^{^{1/_{2}}}}\\[6pt]&=\frac{(\lambda_a r_g-1)}{\left(\lambda_a^2+1-2r_g\lambda_a\right)^{^{1/_{2}}}}\end{aligned}$$

**Fig. 3:**
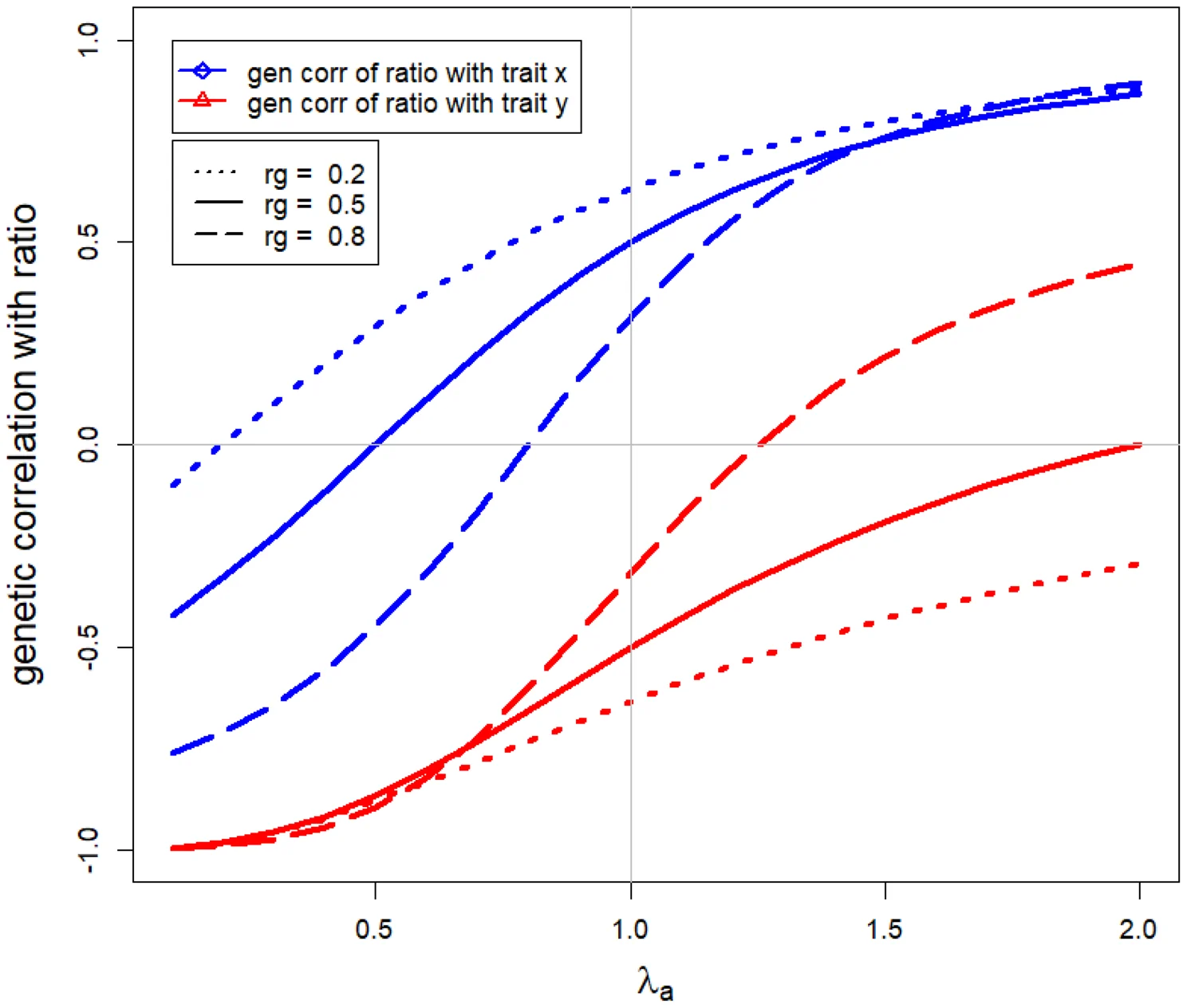
Values for the genetic correlation between a ratio trait and each of the component traits for various values of λ_a_ and genetic correlation between the component traits

The genetic correlation between the ratio trait and trait x is generally positive while the genetic correlation with trait y is mostly negative, as illustrated in Fig. 3. With $$v{p_x}$$ =$$ v{p_y}$$ and $$h_x^2 $$=$$ h_y^2$$, $${\lambda _a} = 1$$, and the genetic correlation between the ratio trait and trait x is equal but of opposite sign to the genetic correlation between the ratio trait and trait y. The genetic correlation is lower or more negative when $${\lambda _a} < 1$$, i.e. when $$h_x^2 $$ < $$ h_y^2$$ or when $$v{p_x}$$ < $$v{p_y}$$ and higher when $$ {\lambda _a} < 1$$, i.e. when $$h_{x }^2 $$>$$ h_y^2$$ or when $$ v{p_x}$$ > $$ v{p_y}$$. When both $$ v{p_x}$$ and $$h_x^2$$ are substantially lower than $$ v{p_y}$$ and $$h_y^2$$, respectively, the value for $${\lambda _a}$$ is very small ($${\lambda _a}$$ < 0.5) and the genetic correlation with the ratio trait could be negative for both component traits while, conversely, when $${\lambda _a}$$ has a high value ($${\lambda _a}$$ > 1.5) the correlation could be positive for both component traits. These genetic correlations are important as they determine the response in the component traits when selecting on a ratio trait.

When the denominator trait y has a higher coefficient of genetic variation, ${\lambda _a} < 1$ and there is relatively more genetic variation in trait y and we can call the ratio “y-dominant” whereas in the reverse, when the numerator trait x as a higher coefficient of genetic variation, ${\lambda _a} > 1$ and the ratio is “x-dominant”. These terms will be relevant when considering the response to selection (see next section). Like the heritability, the values of the correlations of the ratio trait and each of the component traits are not affected by the trait means, and therefore are independent of the units of measure of traits x and y.

### Response to selection on a ratio trait

Using selection index theory and the approximate parameters for a ratio trait, we can predict the response to selection for the component traits when selecting on a ratio trait. Single trait selection on a ratio trait would yield a correlated response in the component traits, calculated as the regression of the ratio on the breeding values of each of component traits x and y.

For selection based on individual phenotype of the ratio, the response for trait x for a selection differential of one standard deviation of the selection criterion will be

9 $${R_x} = \frac{{cov\left( {\frac{x}{y},{a_x}} \right)}}{{\sigma _r^2}}{\sigma _r} = \frac{{r{g_{\frac{x}{y}x}}.{\sigma _{ar}}.{\sigma _{ax}}}}{{{\sigma _r}}} = r{g_{\frac{x}{y}x}}.{h_r}.{\sigma _{ax}}$$

And for trait y

10 $${R_y} = \,\,\frac{{r{g_{\frac{x}{y}y}}.{\sigma _{ar}}.{\sigma _{ay}}}}{{{\sigma _r}}} = \,\,r{g_{\frac{x}{y}y}}.{h_r}.{\sigma _{ay}}$$

**Fig. 4:**
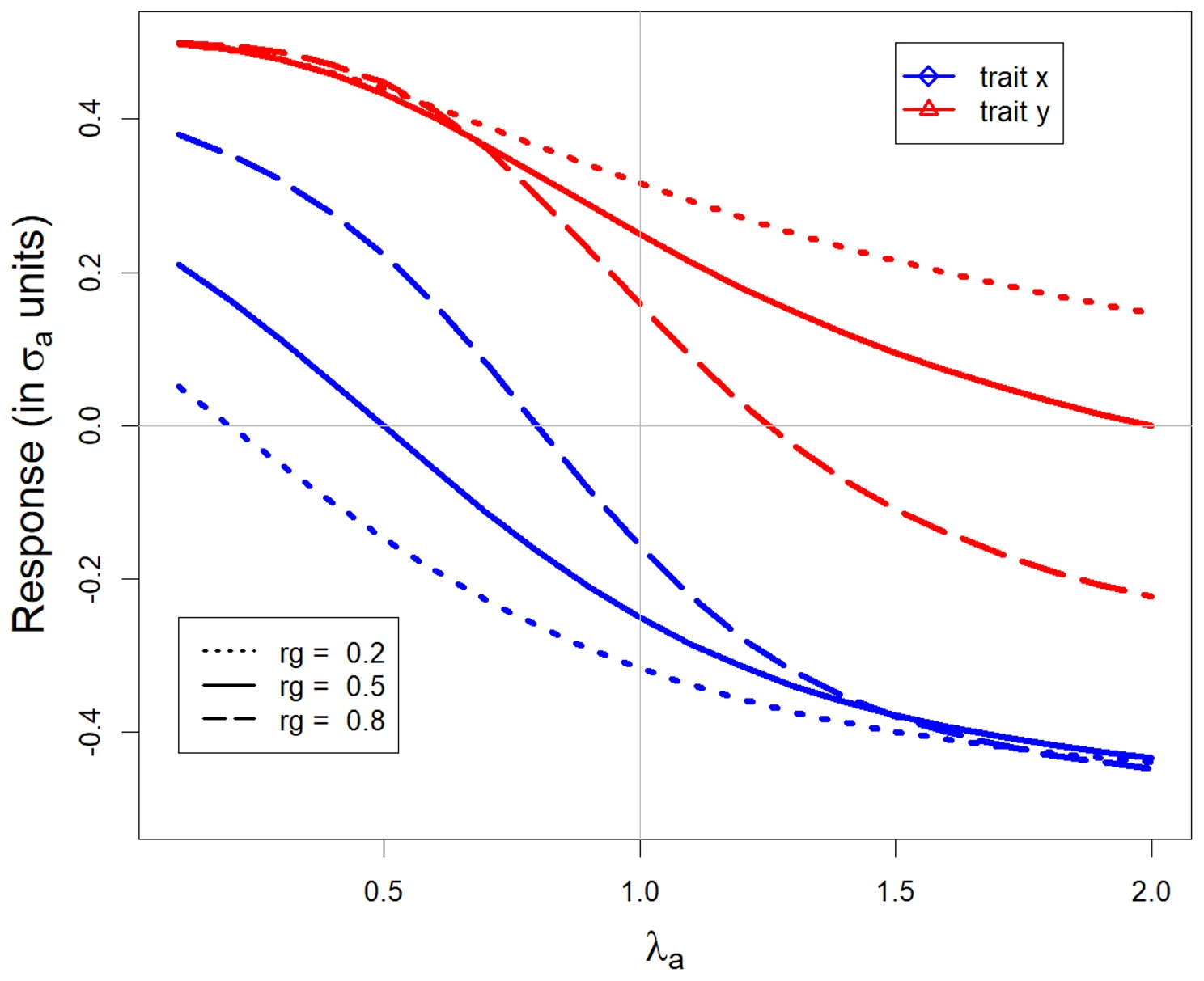
Response to selection in component traits when selecting for a lower ratio (x/y) for various values of λ_a_ and genetic correlation between the component traits

It follows that the ratio of responses in the component traits, in units of genetic standard deviation, is equal to the ratio of the genetic correlations of each of the component traits with the ratio trait. This result holds not only for mass selection, but also for single trait index selection, replacing ${h_r}$ in Eqs. (9) and (10) with the accuracy of the single trait estimated breeding value. Figure 4 illustrates the response to selection for a lower ratio. The figure is very similar but reverse to Fig. 3.

Note that for $${\lambda _a}$$ = 1, the response ratio will be (1$$ - {r_g})$$/$$({r_g} - 1)$$ which is always −1. In other words, with equal coefficient of genetic variation, the absolute value of the response in units of $${\sigma _a}$$ will be equal for the two component traits. If selecting for lower ratios, the response for the numerator trait will be negative while the response for the denominator trait will be positive.

If ${\lambda _a} <$1 and when selecting for a lower ratio, the response for trait y in will always be positive and the absolute value of the response (in ${\sigma _a}$ units) in trait y will always be larger than that in trait x. The response in trait x will also be positive if ${\lambda _a}$ < ${r_g}$, which is more likely for high values of ${r_g}$.

Conversely, if ${\lambda _a} >$1, then selection for a lower ratio will result in a negative response for trait x, and the absolute value of the response (in ${\sigma _a}$ units) in trait x will always be larger than the response in trait y. The response in trait y could become negative if ${\lambda _a}.{r_g}$ > 1, which again is more likely for high values of ${r_g}$. The response in trait x will be equal to zero if ${\lambda _a} = {r_g}$ and the response for trait y will be zero if ${\lambda _a} = 1/{r_g}$ (Fig. 3).

The actual trait responses will also depend on the value of ${\sigma _a}$ which is equal to $va.{\rm{\mu }}$. For example, when selecting for a lower ratio and with ${\lambda _a}$ = 1, the response ratio will be $- {\sigma _{ax}}$ /${\sigma _{ay}}$, which can also be written as $- v{a_x}.{\mu _x}$ /$v{a_y}.{\mu _y}$. So, for ${\lambda _a}$ = 1 the ratio of response for traits x and y will then be equal to $- {\mu _x}$ / ${\mu _y}$. The response will be lower for trait x and less positive for trait y if ${\lambda _a}$ > 1 and it will be lower for both traits if ${\lambda _a}$ < 1 (Fig. 4).

### Selecting on a ratio trait in a multiple trait index

In practical breeding programs, selection is rarely for a single trait such as a ratio trait. A ratio trait can be included in a multi-trait selection index. The parameters required are the variance and heritability of the ratio trait and the phenotypic and genetic correlation between the ratio traits and other traits, including the component traits. Most of these parameters were derived in the previous section.

The correlation between the ratio trait and a third trait can be derived using the same approximation by Pearson [10] as in Eq. (4). For the phenotypic correlation between ratio x/y and trait z we replace the variables for *x*_*1*_, *x*_*2*_, *x*_*3*_ and *x*_*4*_ by x, z, y and 1, respectively, such that

11 $$r{p_{{x \over y},z}} \cong {{r{p_{x,z}}v{p_x}v{p_z} - r{p_{y,z}}v{p_z}v{p_y}} \over {{{\left({vp_x^2 + vp_y^2 - 2r{p_{xy}}v{p_x}v{p_y}} \right)}^{{{}^{1}\!/_{2}}}}{{\left({vp_z^2} \right)}^{{{}^{1}\!/_{2}}}}}}$$

12 $$r{g_{{x \over y},z}} \cong {{r{g_{x,z}}v{a_x}v{a_z} - r{g_{y,z}}v{a_z}v{a_y}} \over {{{\left({va_x^2 + va_y^2 - 2r{g_{xy}}v{a_x}v{a_y}} \right)}^{{{}^{1}\!/_{2}}}}{{\left({va_z^2} \right)}^{{{}^{1}\!/_{2}}}}}}$$

### A ratio trait in the breeding objective

A single trait breeding objective defined as a ratio x/y is equivalent to a linear objective with economic weights μ_y_ and -μ_x_ for the component traits x and y, respectively. This was shown by Lin [4] but a different and perhaps easier way to show this is to take the derivatives of each of the component trait if the objective function is equal to μ_x_ /μ_y_, as ${{\partial \left({{{{\rm{\mu x }}} \over {{\rm{\mu y }}}}} \right)} \over {\partial x}}$= –μ_y_^−1^ and ${{\partial \left({{{{\rm{\mu x }}} \over {{\rm{\mu y }}}}} \right)} \over {\partial y}}$= –μ_x_μ_y_^−2^. Multiplying both weights by μ_y_^2^ gives weights μ_y_ and -μ_x_ for traits x and y, respectively.

The economic weights for a multiple trait breeding objective that includes a ratio trait as well as one of the component traits are more difficult to derive. Assume first a breeding objective based on just the component traits. Based on a profit equation *p* = v_x_ μ_x_ + v_y_ μ_y_ + Constant, we can derive economic weights as partial first derivatives of the profit equation towards each trait mean, giving economic weights v_x_ and v_y_ for traits x and y, respectively. The breeding objective based on component traits is thus

13 $${H_1} = {v_1}\,{a_x} + {v_{2.}}\,{a_{y,}}$$

where a_i_ is the breeding value for trait i and v_i_ is the economic weight of trait i.

When a ratio trait is included in the breeding objective together with component trait y, the breeding objective is

14 $${H_2} = {v_3}\,{a_{x/y}} + {v_4}\,{a_y}$$

The economic weights are a function of the economic weights of the component traits, as used in [2]. The economic weight for the ratio trait v_3_ is the value of one unit change in the ratio, given that the component trait y in the objective does not change. A one unit change in the ratio ${{{\rm{\mu x }}} \over {{\rm{\mu y }}}}$+ 1 is equivalent to ${{{\rm{\mu x }}} \over {{\rm{\mu y }}}}$+ ${{{\rm{\mu y }}} \over {{\rm{\mu y }}}}$= (${{\rm{\mu }}_x}$+ ${{\rm{\mu }}_y}$)/ ${{\rm{\mu }}_y}$. Given that the denominator trait y cannot change, it follows that ${{\rm{\mu }}_x}$ must increase by ${{\rm{\mu }}_y}$ units to be equivalent to a one unit change in the ratio. Therefore, the economic value of one unit change of the ratio trait is the value of ${{\rm{\mu }}_y}$ units of change of the numerator trait: v_3_ = ${{\rm{\mu }}_y}$.v_x_.

Note that the economic weight of trait y must change as well, as it will have to reflect the change of ${{\rm{\mu }}_y}$ by one unit, given that ${{{\rm{\mu x }}} \over {{\rm{\mu y }}}}$ does not change. Therefore, a unit of change in ${{\rm{\mu }}_y}$ must be accompanied by a certain amount of change in ${{\rm{\mu }}_x}$, which we will call z_x_, such that ${{{\rm{\mu x }}} \over {{\rm{\mu y }}}}$ is unchanged. We can write this as (${{\rm{\mu }}_x}$+ z_x_)/(${{\rm{\mu }}_y}$+1) which must be equivalent to ${{{\rm{\mu x }}} \over {{\rm{\mu y }}}}$, therefore ${{\rm{\mu }}_y}$ (${{\rm{\mu }}_x}$ + z_x_)/(${{\rm{\mu }}_y}$+1) = ${{\rm{\mu }}_x}$, and we can solve for z_x_ = ${{\rm{\mu }}_x}$/${{\rm{\mu }}_y}$. Therefore, one unit of change of y must be accompanied by a ${{\rm{\mu }}_x}$/${{\rm{\mu }}_y}$ change in trait x, giving an adjusted economic weight for y as v_4_ = v_y_ + (${{{\rm{\mu x }}} \over {{\rm{\mu y }}}}$).v_x_.

When a ratio trait is included together with component trait x the breeding objective is

15 $${H_3} = {v_5}\,{a_{x/y}} + {v_6}\,{a_x}$$

The economic weights are again a function of the economic weights of the component traits.

The economic weight for the ratio trait v_5_ is the value of one unit change in the ratio, given that the component trait x in the objective does not change. A one unit change in the ratio ${{{\rm{\mu x }}} \over {{\rm{\mu y }}}}$ + 1 is equivalent to ${{{\rm{\mu x }}} \over {{\rm{\mu y }}}}$ + ${{{\rm{\mu y }}} \over {{\rm{\mu y }}}}$ = (${{\rm{\mu }}_x}$+ ${{\rm{\mu }}_y}$)/ ${{\rm{\mu }}_y}$. Given that the numerator trait x cannot change, it follows that ${{\rm{\mu }}_y}$ must change. We can write the new ratio mean as (${{\rm{\mu }}_x}$+ ${{\rm{\mu }}_y}$)/ ${{\rm{\mu }}_y}$ = ${{\rm{\mu }}_x}$/{(${{\rm{\mu }}_x}$/(${{\rm{\mu }}_x}$+${{\rm{\mu }}_y}$)) ${{\rm{\mu }}_y}$}. Therefore, one unit change in the ratio must be equivalent to a change in the mean of trait y of {(${{\rm{\mu }}_x}$/(${{\rm{\mu }}_x}$+${{\rm{\mu }}_y}$)) ${{\rm{\mu }}_y}$} -${{\rm{\mu }}_y}$ = ${{\rm{\mu }}_x}{{\rm{\mu }}_y}$/(${{\rm{\mu }}_x}$+${{\rm{\mu }}_y}$) - $({{\rm{\mu }}_x}$+${{\rm{\mu }}_y}$) ${{\rm{\mu }}_y}$/(${{\rm{\mu }}_x}$+${{\rm{\mu }}_y}$) =(${{\rm{\mu }}_x}{{\rm{\mu }}_y}$- ${{\rm{\mu }}_x}{{\rm{\mu }}_y} - {{\rm{\mu }}_y}{{\rm{\mu }}_y}$) /(${{\rm{\mu }}_x}$+${{\rm{\mu }}_y}$) =$- \mu _y^2$ /(${{\rm{\mu }}_x}$+${{\rm{\mu }}_y}$) units of trait y, therefore v_5_ = [-$\mu _y^2$ /(${{\rm{\mu }}_x}$+${{\rm{\mu }}_y}$)].v_y_.

Note that the economic weight of x must change as well, as it will have to reflect change of ${{\rm{\mu }}_x}$ by one unit, given that ${{{\rm{\mu x }}} \over {{\rm{\mu y }}}}$ does not change. Therefore, a unit of change in ${{\rm{\mu }}_x}$ must be accompanied by a certain amount of change in ${{\rm{\mu }}_y}$, which we will call z_y_, such that ${{{\rm{\mu x }}} \over {{\rm{\mu y }}}}$ is unchanged. We can write this as (${{\rm{\mu }}_x}$+ 1)/(${{\rm{\mu }}_y}$+ z_y_) which must be equivalent to ${{{\rm{\mu x }}} \over {{\rm{\mu y }}}}$, therefore ${{\rm{\mu }}_y}$ (${{\rm{\mu }}_x}$+1)/(${{\rm{\mu }}_y}$+ z_y_)=x, and we can solve for z_y_ =${{\rm{\mu }}_y}$/${{\rm{\mu }}_x}$. Therefore, one unit of change of ${{\rm{\mu }}_y}$ must be accompanied by a ${{\rm{\mu }}_y}$/${{\rm{\mu }}_x}$ change of trait y, giving an adjusted economic weight for trait x as v_6_ = v_x_ + (${{\rm{\mu }}_y}$/${{\rm{\mu }}_x}$).v_y._

### Case study example

A brief numerical example based on beef cattle data is presented to illustrate the theory. The example is taken from [9], who estimated parameters for Feed Intake (FI), Average Daily Gain (ADG) and Feed Conversion Rate (FCR) in Australian beef cattle. Estimates were obtained from measuring Angus steers in the feedlot at an age between 500 and 600 days with trait parameters summarized in Table 1. For this study, economic values for FI and ADG were assumed to be -$60/kg/day and 750$/kg/day, as derived from a simple economic model. Examples of multiple trait index selection were predicted using selection index theory as implemented in MTINDEX [11].

**Table 1:** Phenotypic and genetic parameters for the component traits in the case study example

Component trait Parameter^2^	**Component Traits**^**1**^			Feed Conversion Ratio		
	Feed Intake		Average Daily Gain	Ratio trait Parameters^3^	Predicted^4^	Estimated^5^
Mean	14.9		1.59	Mean	9.7	9.78
$\upsilon {p_i}$	0.13		0.22	${\lambda _p}$	0.59	na
$\sigma {p_i}$	1.94		0.35	$\sigma _p^2$	3.47	3.54
$\sigma {a_i}$	1.44		0.2	$\sigma _a^2$	0.68	0.71
${h^2}$	0.55		0.33	${h^2}$	0.20	0.20
$\upsilon {a_i}$	0.1		0.13	${\lambda _a}$	0.77	na
${r_{p \left({FI,ADG} \right)}}$		0.45		${r_{p \left({FCR, FI} \right)}}$	0.16	0.05
				${r_{p \left({FCR, ADG} \right)}}$	−0.81	−0.78
${r_{g \left({FI,ADG} \right)}}$		0.72		${r_{g \left({FCR, FI} \right)}}$	0.06	−0.12
				${r_{g \left({FCR, ADG} \right)}}$	−0.65	−0.69

^1^ Parameters from [9]

^2^$\upsilon {p_i}$ = coefficient of variation of trait i,$\sigma {p_i}$and$\sigma {a_i}$are phenotypic and additive genetic standard deviation,${h^2}$is heritability and r_p_ and r_g_ are phenotypic and genetic correlations

^3^${\lambda _p}$is the ratio of coefficient of variation,$\sigma _p^2$and$\sigma _a^2$are phenotypic and additive genetic variance,${\lambda _a}$is the ratio of coefficient of genetic variation

^4^ Predicted parameter values for feed conversion based on the theory described under methods

^5^ Estimated parameter values for feed conversion based on [9]

## Results

The parameters for the component traits Feed Intake (FI) and Average daily gain (ADG) in the examples were estimated by [9] and are summarised in Table 1. From these component trait parameters, we can predict parameters for Feed Conversion Rate (FCR), which is the ratio FI/ADG. These predicted parameters for FCR can be compared with the parameter values estimated by Torres-Vázquez et al. [9] (Table 1).

**Fig. 5:**
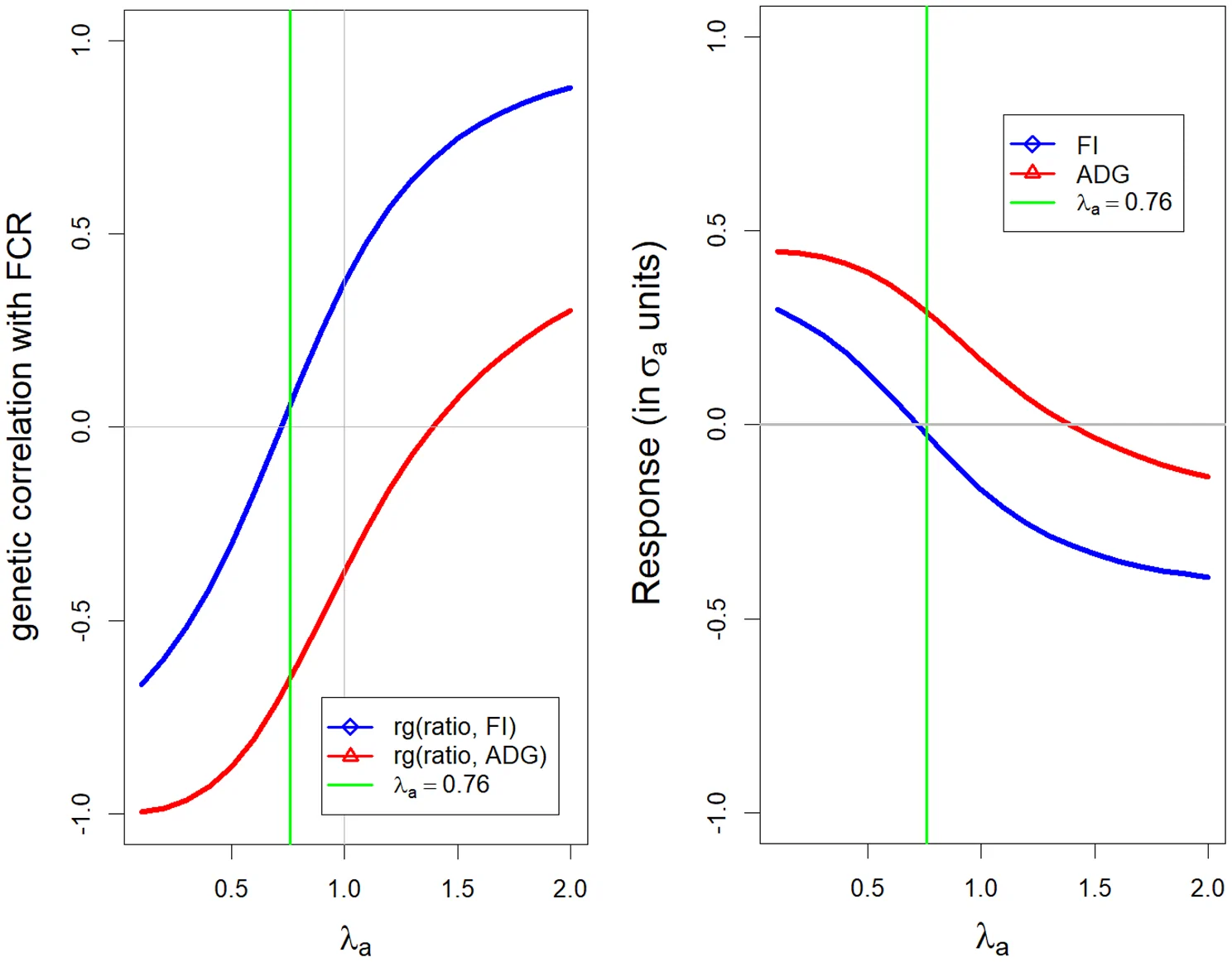
Genetic correlation between FCR and component traits and response in component traits when selecting for lower FCR for different values of ${\lambda _a}$

Overall, the genetic and phenotypic parameters for FCR that were estimated agree closely with the predicted values based on component trait parameters. This includes the prediction of the heritability of the ratio trait to be considerably lower than the heritability of either of the component traits, which is due to the phenotypic correlation between the component traits being lower than the genetic correlation (Fig. 2). Furthermore, the lambda values are lower than 1, meaning that the ratio trait is ‘y-dominant’, and mostly affected by the denominator trait (ADG in this example). This also leads to phenotypic and genetic correlations being much stronger between FCR and ADG than between FCR and FI. The relationship between values of ${\lambda _a}$ and the correlations between ratio and component traits is shown in Fig. 5, assuming a genetic correlation between the component traits of 0.72. The predicted genetic correlations of the ratio FI/ADG with FI and ADG are 0.06 and −0.65, respectively.

The response (in units of σ_a_) to single selection for lower FCR is predicted using Eqs. (9) and (10) and illustrated in Fig. 5. Due to the ratio trait being ‘y-dominant’, the selection response is stronger for the denominator trait, in this case ADG, whereas the response in FI is negligible. Therefore, given the phenotypic and genetic parameters, selection for lower FCR will mostly increase ADG and have limited effect on FI.

**Fig. 6:**
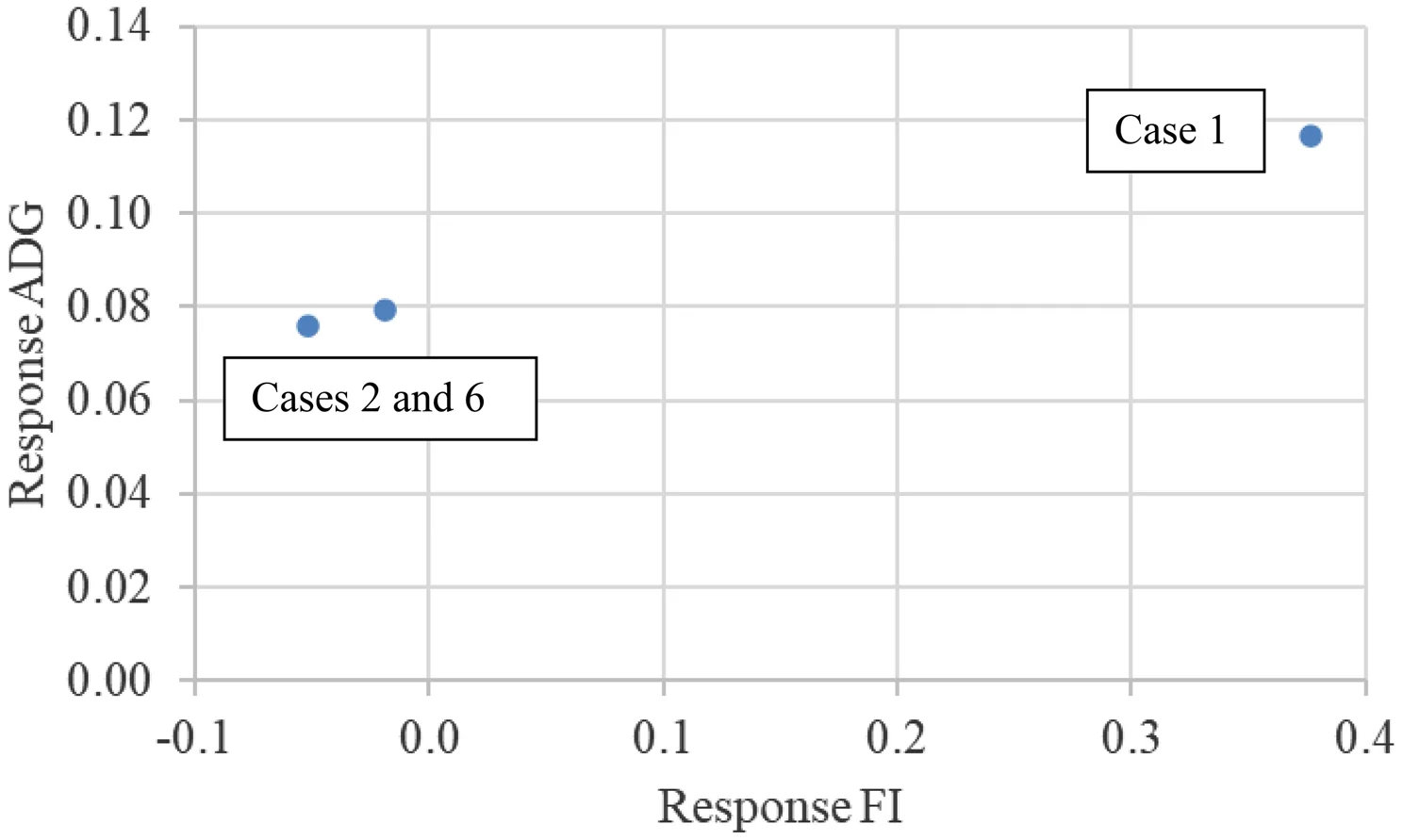
Response to selection in FI and ADG with selection on a linear index (case 1), FCR (case 2) or FCR and ADG (case 6)

The outcomes of various multiple-trait selection strategies are shown in Table 2. These strategies involve different combinations of the ratio trait (FCR) and its component traits (FI and ADG) as selection index criteria and as components of the breeding objective. Selection criteria for each trait are estimated breeding values (EBVs), using information on all selection criteria traits. The information assumed known for each trait is equal to ten progeny, which mimics an EBV accuracy of a moderately accurate genomic test (accuracies for FI, ADG and FCR of ~0.8, 0.7 and 0.6, respectively). Cases 1–4 in Table 2 refer to a breeding objective that includes both component traits. Results show that selection on a ratio (case 2) gives a very different response compared with selection on a linear index with the component traits (case 1) (Fig. 6). Selection directly on the component traits as in case 1 gives an optimal economic response by increasing ADG while accepting also an increase in FI. The main emphasis on ADG can be explained by the higher value of the trait, measured as dollar value of one genetic standard deviation; −86 for FI and +151 for ADG.

**Table 2:** Comparison of different selection strategies, with various combinations of the ratio trait (FCR) and its component traits (FI and ADG) as selection index criteria and as components of the breeding objective

Case	Breeding objective	Economic value FI (v1)	Economic value ADG (v2)	SD BrObj	Selection Criteria^1^	Response FI kg/day	Response ADG kg/day	Response FCR kg FI/kg ADG	Response Profit^2^
1	v_1._FI + v_2_.ADG	-$ 60.00	$ 750.00	$ 106.94	FI, ADG	0.377	0.116	−0.450	$ 64.75
2	v_1._FI + v_2_.ADG	-$ 60.00	$ 750.00	$ 106.94	FCR	−0.052	0.076	−0.479	$ 59.98
3	v_1._FI + v_2_.ADG	-$ 60.00	$ 750.00	$ 106.94	FCR, ADG	0.377	0.116	−0.450	$ 64.75
4	v_1._FI + v_2_.ADG	-$ 60.00	$ 750.00	$ 106.94	FCR, FI	0.377	0.116	−0.450	$ 64.75
5	- FCR	-$ 1.00			FCR	−0.052	0.076	−0.479	$ 59.98
6	- FCR	-$ 1.00			FI, ADG	−0.018	0.079	−0.480	$ 60.67
7	v_1._FI + v_2_.ADG	-$ 1.59	$ 14.9		FCR, ADG	−0.018	0.079	−0.480	$ 60.67
8	- FCR	-$ 1.00			FI, FCR	−0.018	0.079	−0.480	$ 60.67
9	FCR + ADG	-$ 95.40	$ 187.74	$ 106.94	FCR, ADG	0.377	0.116	−0.450	$ 64.75
10	FCR + ADG	-$ 95.40	$ 187.74	$ 106.94	FCR, FI	0.377	0.116	−0.450	$ 64.75
11	FCR + ADG	-$ 95.40	$ 187.74	$ 106.94	FI, ADG	0.377	0.116	−0.450	$ 64.75

^1^ Selection criteria traits are included as estimated breeding values estimated based on multiple trait information. The information assumed for each trait is equivalent to having 10 progeny measured for each trait

^2^ Profit response is always calculated using the response and the economic value for the component traits FI and ADG

Selection on FCR reduces FI and improves ADG. The response in units of genetic standard deviation are −0.04 for FI and 0.38 for ADG, which is expected with a ${\lambda _a}$ value of 0.76 (Fig. 5). The relatively larger response (in units of σ_a_) in ADG can be explained by its higher coefficient of genetic variation. Although these responses are in the desirable direction for both traits, the overall economic response is not optimised and 7.4% lower than the optimal response. The response in FCR in case 2 is higher (more negative) than in case 1. However, economically it is more valuable to increase the denominator trait, so while case 2 gives more response for FCR, it is not economically optimal.

Cases 3 and 4 include FCR as a selection criterion alongside one of the component traits. The results show that the selection response is identical to case 1. In other words, selecting on the ratio trait alongside one of the component traits is equivalent to selecting on a linear index with the two component traits

Cases 5, 6 and 8 refer to a breeding objective where FCR is the only breeding objective trait. When selecting only on FCR as in case 5, the response is the same as in case 2 as single trait selection is not dependent on the breeding objective. Including one of the component traits as an additional selection criterion gives a slightly higher response as the FCR EBV would be estimated with slightly higher accuracy (cases 6 and 8). Note that this increase in accuracy is the same in both cases and not dependent of which component trait is included alongside the ratio trait. Case 7 is included to demonstrate that a breeding objective with just one ratio trait is equivalent to a breeding objective with the component traits weighted by the trait means, with weights μ_y_ and -μ_x_ for traits x and y, respectively.

Cases 9–11 refer to breeding objectives that include the ratio trait as well as one of the component traits. Results are identical to cases 1, 3 and 4. Therefore, including a ratio trait in the breeding objective can give identical results as a linear breeding objective with the two component traits, provided that economic values are adjusted. The ratio trait has economic value $${{\rm{\mu }}_y}$$.v_x_ = 1.59.(-$60) = -$95.4 while the economic value for ADG is adjusted according to v_y_ + ($${{\mu { _x}} \over {{\mu _y}}}$$).v_x_ = $ 750 + (14.9/1.59).(-$ 60) = $187.74.

## Discussion

Single trait selection on a ratio of component traits leads to a selection response that is determined by the ratio of coefficients of genetic variation of the component traits (λ_a_ = νa_x_/νa_y_). If the denominator trait has a higher νa_i_, then most of the response will be focused on reducing the denominator trait, and vice versa. While this gives a higher response for the ratio, it is not necessarily the most profitable response. A ratio can be altered by changing either the numerator or the denominator trait, i.e. in the example by either reducing FI or by increasing ADG. Because ADG has a higher coefficient of genetic variation, selecting on a ratio will mostly change ADG with a limited response for FI. The economic optimum is to increase ADG even further with a correlated positive response for feed intake being economically offset by more genetic gain for ADG. If feed costs were higher, an optimal response might see a decrease in FI with less genetic change in ADG. The example shows that maximizing response in a ratio is not always economically most optimal. However, as also demonstrated in this paper, if the component trait is used in the index alongside the ratio trait, the index becomes equivalent to a linear index with the two component traits. The same can be shown to hold more generally when additional traits are added to the index (data not shown).

The dependency of the ratio trait on the parameters of the component traits leads to an equivalence between these indices, i.e. either an index with both component traits, or an index with the ratio trait and one of the component traits. If both component traits are included in the index alongside their ratio trait, the ratio trait becomes redundant and the index weight of one of the component traits would be zero.

The equivalence between an index with a ratio trait and an index with component traits is a property that has also been shown for residual traits, e.g. an index including residual feed intake with ADG would be equivalent to an index including Feed intake alongside ADG [2, 3]. A mathematical proof of the equivalence is easier shown for residual traits as these are linear combinations of the component traits, and variances and covariances of a sum of variables are easy to derive. However, ratio traits cannot be written as linear combinations of component traits and variances and covariances of products (or ratios) of variables are less straightforward, as shown in this paper. An attempt of a mathematical proof of the equivalence of these indices has therefore not been shown, but it is important to note some consequences of this equivalence, using selection index theory. Assume a vector *x* with information sources to estimate genetic merit, with var(*x*) = P, and a breeding objective trait with breeding value *a*_*i*_. The breeding value *a*_i_ can be estimated based on information in *x*, by regression as *â*_i_ = P^−1^G_i_*x*, where G_i_ = cov(*x*, *a*_i_). The variance of *â*_i_ is var(*â*_i_) = G_i_^’^P^−1^G_i_ and the accuracy is var(*â*_i_)/var(*a*_i_). Now assume Index A with information sources on the two component traits in *x*, and Index B with equivalent sources of information on the ratio trait and only one of the component traits in x^*^. The corresponding selection index matrices are P and G_i_ for index A and P^*^ = var(x^*^) and G_i_^*^ = cov(x^*^, *a*_i_) for index B. Since both indices give the same results, the accuracy of the estimated breeding values of the breeding objective traits must be the same, i.e. G_i_^’^P^−1^G_i_ = G_i_^*’^P^*-1^G_i_^*^. It should be noted that unlike an index including a residual trait, indices for ratio traits maybe not strictly equivalent, but rather they are similar to indices for component traits, as parameters for ratio traits are approximated. Moreover, Gunsett [5] pointed out that when selecting on a ratio, the pressure placed on component traits is dependent on selection intensity, making prediction of genetic change less predictable. This could be further explored for indices that contain ratio traits as well as one of the component traits.

Using a ratio trait as a single objective trait will generally not give an economically optimal response, as it implies that the weights for the component traits in the objective are proportional to μ_y_ and -μ_x_, which are almost invariably not the same as the actual economic weights. Using a breeding objective that includes both a ratio trait and one of the component traits would give an equivalent result to a linear objective with both component trats if economic values are appropriately derived. However, this study showed that deriving economic values for a component trait as well as a ratio trait is not trivial because of the mathematical dependency between the two variables.

The results of this study have implications for strategies that select for productivity, feed efficiency and methane intensity, the latter two both ratio traits. The study shows that ratio traits could be included in indices as long as one of the component traits is also included. A disadvantage of using ratio traits as selection criterion is that both phenotypes need to be measured on the same animals. Frequently, one of the component traits will be missing, or the different component traits may be measured on different animals. This poses no problem in a multiple trait genetic evaluation with component traits. In the era of genomic selection and single step genetic evaluation, it is increasingly less important to measure traits on the same animals. For example, there may be one reference population with just feed intake measured while another has only measurements for ADG. Genotyped individuals can then obtain genomically estimated breeding values (GEBV) for each of the component traits, but they cannot have a GEBV for the ratio of those traits based on an analysis of phenotypes of the ratio trait.

Residual traits such as residual feed intake (RFI) or residual methane are sometimes proposed as alternatives to ratio traits. As already pointed out, using residual traits in breeding objectives or indices is statistically equivalent to including the component traits directly, with appropriate adjustments to genetic parameters and economic weights [2]. Defining residual traits at the phenotypic level is less desirable because it requires that both component phenotypes be measured on the same animals. Instead, EBVs for residual traits can be generated from EBVs of the underlying component traits using their estimated variances and covariances. Similarly, it may be possible to derive EBVs for ratio traits based on EBVs for the component traits, although this will not be a simple linear transformation as is the case with residual traits. Whether providing such additional selection tools is beneficial will depend in part on the adoption and understanding of the traits presented under these different options and may warrant further investigation.

A trait like feed conversion ratio is a relatively simple ratio trait. However, in the case of methane traits, methane intensity is often promoted as a desirable trait, which is methane per unit of product output. The latter is not necessarily one trait but could be a function of many traits. For example, in sheep and beef cattle, methane intensity could be expressed per kg of meat produced. But in a production system, the amount of meat produced per breeding female is a function of multiple traits such as growth rate and number of progeny weaned per female per year. Also, the product quality may have to be included in the measure. It would be difficult to derive appropriate economic weights for a ratio trait that depends on multiple underlying component traits. A simple linear index framework maybe more straightforward when dealing with a linear combination of many component traits, each weighted by their economic value and predictions of merit and response depend on the variances and covariances of those traits. Veerkamp [12] discussed how a selection index framework can accommodate a linear function of multiple traits to capture their contributions to feed efficiency. Such a linear multiple trait evaluation system is robust against missing data and can accommodate greater complexity arising from many traits.

## Conclusion

An index including a ratio trait alongside one of its component traits yields equivalent results to a linear index based on the two component traits. Likewise, a breeding objective that includes both a ratio trait and a component trait yields the same selection outcomes as an objective based on component traits alone, provided that the economic values of the component traits are correctly specified. Single-trait selection on a ratio of component traits is not economically optimal, as the selection response is governed by the ratio of the coefficients of genetic variation of the component traits, rather than by their economic values. Similarly, defining a ratio trait as the sole breeding objective implicitly applies weights proportional to the means of the component traits, an outcome that rarely reflects their true economic values.

Beyond theoretical equivalences, the use of ratio traits in practical breeding programs will also depend on additional factors, including the understanding and adoption of efficiency traits by end users. Practical and statistical challenges would also need to be addressed, including how to account for cases where both component traits are not measured on the same individuals, which complicates the use of ratio traits in routine genetic evaluation. One option, analogous to the use of residual traits, would be to perform genetic evaluation based directly on the component traits and to derive ratio (or residual) trait summaries from the EBVs of component traits post-evaluation.

## Data Availability

There is no data used for this paper. Not applicable.
